# Targeting Tyrosine Kinases in Acute Myeloid Leukemia: Why, Who and How?

**DOI:** 10.3390/ijms20143429

**Published:** 2019-07-12

**Authors:** Solène Fernandez, Vanessa Desplat, Arnaud Villacreces, Amélie V. Guitart, Noël Milpied, Arnaud Pigneux, Isabelle Vigon, Jean-Max Pasquet, Pierre-Yves Dumas

**Affiliations:** 1 Institut National de la Santé et de la Recherche Médicale, U1035 Bordeaux, France; 2 Université de Bordeaux, 33076 Bordeaux, France; 3 CHU Bordeaux, Service d’Hématologie Clinique et Thérapie Cellulaire, F-33000 Bordeaux, France

**Keywords:** acute myeloid leukemia, tyrosine kinase, inhibitors, targeted therapy

## Abstract

Acute myeloid leukemia (AML) is a myeloid malignancy carrying a heterogeneous molecular panel of mutations participating in the blockade of differentiation and the increased proliferation of myeloid hematopoietic stem and progenitor cells. The historical “3 + 7” treatment (cytarabine and daunorubicin) is currently challenged by new therapeutic strategies, including drugs depending on the molecular landscape of AML. This panel of mutations makes it possible to combine some of these new treatments with conventional chemotherapy. For example, the FLT3 receptor is overexpressed or mutated in 80% or 30% of AML, respectively. Such anomalies have led to the development of targeted therapies using tyrosine kinase inhibitors (TKIs). In this review, we document the history of TKI targeting, FLT3 and several other tyrosine kinases involved in dysregulated signaling pathways.

## 1. Introduction

Medicine has traveled a long way since the end of the nineteenth century, when blast cells were characterized in the bone marrow of acute myeloid leukemia (AML) patients. In the last 20 years, the treatment of AML has not kept step with knowledge about the molecular abnormalities leading to leukemogenesis, despite intensive academic and industrial research. While the prognostic classification of AML has improved, therapeutic management is still a matter of debate. Currently, targeted therapies including tyrosine kinase inhibitors (TKIs) are regularly used. Others are still in development to achieve better results in this difficult-to-treat disease.

## 2. Kinome and Tyrosine Kinase

Protein kinases play a key role in signal transduction. About 500 human genes encode kinases while more than 1000 are present in the genome of the popular model in plant biology, *Arabidopsis thaliana* [1,2,3]. Among the kinases, tyrosine kinase (TK) phosphorylation was discovered serendipitously [4]. Until now, about 100 TK have been characterized in humans and are distributed between receptor TK (RTK) and cytoplasmic TK [5]. TK activity is required to phosphorylate substrates on tyrosine residues, including autophosphorylation, allowing the recruitment of signaling partners at docking sites. Tyrosine phosphorylation controls the signaling pathways involved in many cellular processes such as cell growth, proliferation, differentiation and metabolism [6]. Deregulation of the expression and/or activity of TK through mutations or other mechanisms leads to a wide range of diseases and cancers. Blocking its enzymatic activity therefore became a quest for pharmaceutical companies [7,8,9,10]. Around 50 kinase inhibitors are currently FDA approved and referenced in http://www.brimr.org/PKI/PKIs.htm, while at least 150 are being investigated in clinical trials [11,12].

## 3. Tyrosine Kinase in Hematopoietic Tissue

Hematopoiesis in adults subsumes all the biological processes that allow hematopoietic stem cells (HSC) to give rise to all blood lineages in the bone marrow and mature blood cell populations. Several TKs play a key role at different steps of hematopoiesis [13]. To date, at least three have been reported to play a role in early hematopoietic stem and progenitor cell (HSPC) growth and differentiation: (i) the macrophage colony-stimulating factor receptor (M-CSFR) [14]; (ii) the stem cell factor receptor (SCFR, KIT) [15]; and (iii) the FMS-like TK 3 (FLT3) [16]. All other hematopoietic growth factors possess receptors without TK enzymatic activity, although most of them recruit cytoplasmic kinases for signaling. These growth factors and cytokines bind to their own receptors, of which many interact with Janus kinases (JAKs) [17,18]. Other cytoplasmic TKs are involved in signaling pathways downstream of these receptors and JAKs. One family is in close vicinity with JAK: the SRC family TK (SFK).

The SFK, which comprises eight members and three kinase-like SRCs, has been shown to be involved in various lineages of hematopoiesis [19,20,21] such as erythroid and megakaryocyte lineages [22,23]. Six SRC TKs are predominantly expressed in blood cells and disruptions of several SRC TKs induce hematologic abnormalities. While the knock-out (KO) of a single *Src* TK gene is not lethal, the KO of the *Src Lyn* gene induces a phenotype of lupus syndrome, suggesting its key role in the control of immune response [24], and HCK single KO associates with extramedullary hematopoiesis. In contrast, double KO can be lethal [25,26]. LYN is also involved downstream of JAK TK and its inhibition prevents CSF1 or G-CSF-induced proliferation [27,28]. LYN also closely interacts with KIT and its molecular inhibition prevents the SCF-induced proliferation of HSPC [29].

Another TK family, the TEC family, including BMX, BTK, ITK, TEC and TXK, is required not only for the development of B and T cells but also for their specific signaling. TEC TKs were identified in hepatocellular carcinoma by screening a cDNA library [30] and they play a major role in immunity [31]. BTK mutations are associated with a decreased affinity towards phosphoinositides and lead to X-linked immunodeficiency in humans and mice. Over 400 BTK mutations have now been described and are spread throughout the gene [32]. Current research is probing the role of BTK and TEC in AML.

SYK and Zap70 TKs are involved in B and T cell signaling, respectively [33,34]. While SYK is present in many tissues, its role in hematopoiesis and hematological disorders has been reported [35]. Mice KO for *Syk* showed B cell deficiency, abnormal hemostasis and embryonic lethality [36,37].

Other TKs such as the FES/FER family are involved in hematopoiesis, but FES does not seem to be a prerequisite for normal myeloid lineage [38]. It may play a redundant function since mice KO for *Fes* showed only slight differences [39]. FES interacts with cytokine receptors, and PU.1 is one of the transcription factors inducing its expression [40].

## 4. Tyrosine Kinase in Acute Myeloid Leukemia

The deregulation of RTK and TK expression and/or activity is a classical hallmark of cancer cells [41]. Deregulation of TK signaling leads to oncogenic signals and has long been identified in malignant hematopoiesis [41,42,43]. The t(9;22), reciprocal translocation giving rise to the Philadelphia chromosome allows expression of a recombinant chimeric TK protein, BCR-ABL1, which constitutively activates many signaling pathways that are usually tightly regulated, leading to chronic myeloid leukemia (CML) [44]. CML is the main TK-dependent cancer because it was the first to be targeted by an FDA-approved therapy, the anti-ABL TKI imatinib.

**Figure 1:**
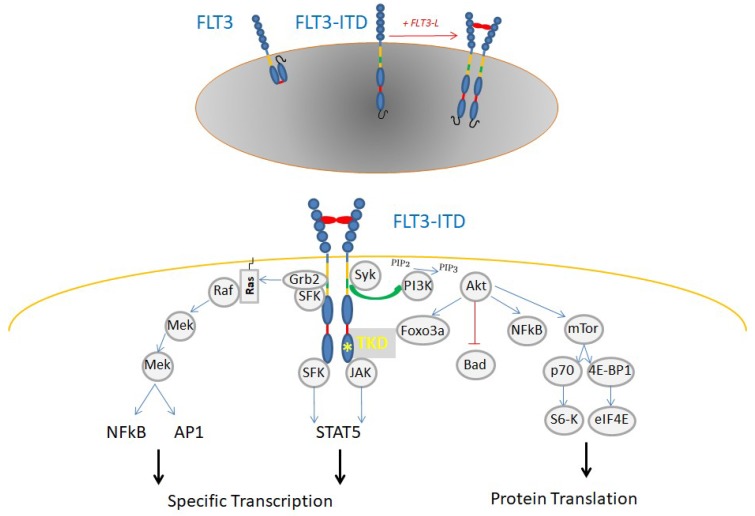
Signaling in AML FLT3-ITD. Like most receptor tyrosine kinases, FLT3 is activated following ligand-induced dimerization and then signals through two major pathways: PI3-kinase/AKT and RAS/RAF/MAP-kinases. Internal tandem duplication by changing structure and localization induces constitutive activation of FLT3 signaling pathway with a large increase in STAT5 activation.

In AML, the FLT3 receptor, which is usually present on HSPC, is frequently overexpressed and mutated at diagnosis through an internal tandem duplication (25%, ITD) or point mutation in the TK domain (7%–10%, TKD) (Figure 1). Both induce a constitutive signaling associated with the sustained activation of STAT5, resulting in AML proliferation and survival. Correlated to its overexpression, its activation through ligand binding induces signal activation, depicted in Figure 1. An increase in FLT3-ITD ratio at relapse suggests an advantage for the mutated clone and is associated with a poor outcome in AML patients [45]. TKs are also involved in other hematopoietic disorders such as myeloproliferative neoplasms (MPN) [46,47].

AML represents 3% of cancers and 25% of leukemias [48]. It is a heterogeneous hematopoietic malignancy characterized by a specific molecular landscape. Beyond the recurrent chromosomal abnormalities, a large panel of mutations has been identified and generally comprises eight functional categories: (i) signaling pathways that include genes coding for TK proteins (FLT3, c-KIT, (RAS, PTPN11); (ii) transcription factors (RUNX1, CEBP); (iii) spliceosome complex (SRSF2, SF3B1, U2AF1, ZRSR2); (iv) cohesin complex (STAG2, RAD2); (v) epigenetic modifiers with chromatin-modifying genes (ASXL1, ASXL2, EZH2, MLL) and (vi) genes involved in DNA methylation (DNMT3A, TET2, IDH1 and IDH2); (vii) tumor suppressor genes (TP53, PTEN, PHF6); and (viii) NPM1 mutations. NPM1 mutations are isolated because the mechanisms underlying their leukemogenic pathways are multi-faceted (genomic instability, MYC activation, HOX overexpression, ARF relocation, etc.) [49]. It is commonly accepted that, compared to solid tumors, AML has few mutations, suggesting that other factors, including epigenetic ones, contribute to leukemogenesis.

TK mutations in AML involve mainly FLT3 (30%), c-KIT (5%) and JAK2 (2%) and to a lesser extent JAK1, JAK3 and CSF3R. However, TK negative regulators such as PTPN11 (15%) or CBLC and to a lesser extent PTPN14 and PTPRT are also involved. Mutations affecting RAS proteins, which are downstream of multiple RTKs, are beyond the scope of this review. TKs are also frequently deregulated in AML cells, with the overexpression of unmutated proteins able to activate pro-oncogenic signaling. In AML, SYK is a potential target, as its inhibition led to the differentiation of AML cells in vitro and in vivo [50]. SYK is an interesting target since its overexpression promotes AML transformation and resistance to treatment [51]. In addition, its role in lymphoid malignancies led to the development of the TKIs that are currently being tested in clinical trials and transposed to AML [52].

The FLT3 mutation is by far the most frequent TK mutation in AML, leading to the development of a large panel of TKIs. FLT3 encodes a class III RTK that is well expressed in HSPCs and activates the PI3K/AKT and MAPK pathways upon ligand binding. This RTK family also includes KIT, CSFR and PDGFR, comprising an extracellular domain containing five immunoglobulin domains. FLT3-ITD is the most frequent mutation in AML, involving 4 to 400 base-pair insertions [53,54]. FLT3-ITD is also present in 10% of pediatric AML, 1% of myelodysplastic syndrome (MDS) and 3% of acute lymphoblastic leukemia (ALL). Although the FLT3-ITD mutation is a late event in leukemogenesis, it is an important target for the disease [55]. FLT3-ITD carries a poor prognosis in adult AML patients [56].

## 5. Tyrosine Kinase Inhibitors

**Figure 2:**
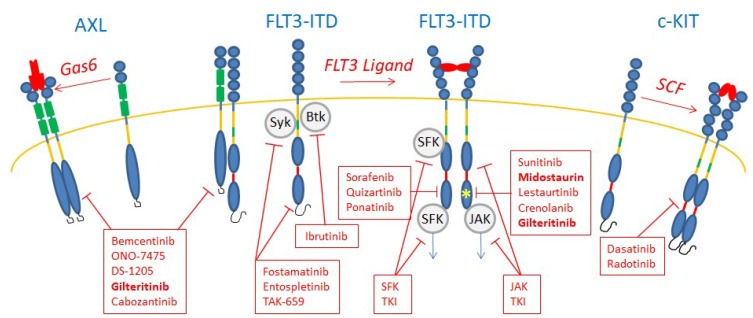
Tyrosine kinase inhibitors in FLT3-ITD AML. Midostaurin and gilteritinib are shown in bold as the only two FDA-approved TKIs. All the others are under development and most are being tested in clinical trials. Those still in preclinical development are not presented.

The chemical classification of kinase inhibitors has been recently reviewed by R. Roskoski Jr. through the structure of the enzyme-bound antagonist complex. Type I inhibitors bind to the active and inactive protein kinase conformation, type II bind to the inactive protein kinase conformation, type III and IV are allosteric, type V are bivalent and type VI bind covalently to their target [11]. Table 1 shows a panel of TKIs used in AML or in clinical trials and Figure 2 shows targeted TKs.

### 5.1. FLT3 Tyrosine Kinase Inhibitors

Type I inhibitors include midostaurin, gilteritinib and crenolanib, and have activity against the FLT3-ITD and -TKD mutations, while type II inhibitors, which include sorafenib and quizartinib, do not have activity against TKD mutations, as the latter favor the active (DFG-in) protein conformation. Pan TKI first-generation inhibitors such as midostaurin and sorafenib have marginal single-agent activity, even if this postulate may be challenged for allogenic transplant maintenance therapy [57]. Conversely, several FLT3 TKIs such as quizartinib, crenolanib and gilteritinib have single-agent activity that leads to complete or near-complete remission, supporting the rationale for the combination of these agents with cytotoxic chemotherapy. Other studies evaluating quizartinib and gilteritinib in association with various chemotherapy regimens are ongoing, while both TKIs have demonstrated an overall survival (OS) benefit as monotherapy in refractory and relapse (R/R) AML. They are therefore the focus of new development strategies in FLT3-mutated AML. The emergence of resistance is expected through various mechanisms, including intrinsic mechanisms such as the activation of bypass signaling pathways and the activation loop or gatekeeper mutations and extrinsic mechanisms, including cell-to-cell interactions and the secretion of various cytoprotective factors [58,59].

Lestaurtinib (CEP-701 Type I) is a first-generation FLT3 TKI, which inhibits JAK2 WT and mutated in MDS cells. Lestaurtinib is an orally available TKI targeting several RTKs and inhibiting constitutively active FLT3 [60]. It has been used firstly in refractory/relapsed (R/R) AML and it is proposed in newly diagnosed AML [61]. Results are a transient decrease of blast in bone marrow. Both at diagnostic and in R/R AML, it induces a decreased blast count, but responses are short-lived.

Sorafenib (BAY43-9006 Type II) is a first-generation FLT3 TKI and a multikinase inhibitor (RAF, PDGFR, VEGFR, c-KIT, FLT3). As a single agent, sorafenib can induce remission in relapsed FLT3-ITD AML by the downregulation of MCL1 and the upregulation of BIM [62,63]. The most interesting results have recently been shown in the post-HSCT (hematopoietic stem cells transplant) setting (SORMAIN study), probably through immune pathways in addition to the classical pathways [64,65].

Midostaurin (PKC412 Type I) is also a first-generation FLT3 TKI, initially developed to target protein kinase C (PKC). As a type I TKI, it inhibits FLT3-ITD and TKD in vitro and in vivo [66]. It was the first TKI against FLT3 to be FDA approved in April 2017 (Figure 2). The randomized phase III RATIFY study evaluated chemotherapy with or without midostaurin for patients with newly diagnosed FLT3-mutated AML and showed an OS benefit in the midostaurin arm [67].

Sunitinib (SU11248 Type I) is a multitargeted TKI (FLT3, PDGFR, VEGFR, c-KIT). It exerts an equal block on FLT3-ITD and TKD [68]. A phase I study showed better responses in FLT3-mutated AML, although of short duration [69]. In a phase I/II trial, it synergized with cytarabine/daunorubicin in FLT3-ITD but not FLT3 WT AML, which are thought to be the clone which relapses [70].

Quizartinib (AC220, Type II) is a second-generation FLT3 TKI with high selectivity for FLT3 (IC50 ≈ 1 nM), although having activity against c-KIT and PDGFR but with a 10-fold IC50 [71,72]. In clinical trials, it was well tolerated and demonstrated efficacy in improving clinical outcomes as a single agent in R/R AML patients (QUANTUM-R study) [73,74,75,76].

Crenolanib (CP868596, Type I) inhibits FLT3-ITD and -TKD [77,78]. In a phase I study, it demonstrated activity in R/R AML patients and led to 39% complete remission [79,80]. Used as a single agent, a recent study reported bypass mechanisms through adding mutations affecting NRAS and IDH2 [81]. Phase III clinical trials are underway in newly diagnosed FLT3-mutated AML versus midostaurin (NCT03258931) and in R/R FLT3-mutated AML in association with chemotherapy versus chemotherapy alone (NCT03250338).

Gilteritinib (ASP2215, Type I) is an FLT3/AXL inhibitor that also has activity against ALK TK. A phase I/II trial in R/R AML demonstrated a good overall response rate in FLT3-ITD and -TKD AML and less in unmutated FLT3 AML [82,83]. Several trials are underway to test gilteritinib as induction/consolidation treatment. A recent study reported resistance against gilteritinib in AML through TK-independent pathways [84]. In clinical trials, it is well tolerated and demonstrated efficacy in improving clinical outcomes as a single agent in R/R AML patients (ADMIRAL study) [85].

FF-10101 is an irreversible TKI through covalent binding to cyst 695 of FLT3 [86,87]. It shows activity against FLT3-mutated AML cells in vitro and is currently being studied in patients with R/R AML in a phase 1/2 study (NCT03194685). Some FLT3-TKIs are currently in preclinical development (e.g., G-749, TTT-3002), others are in early clinical development (e.g., AKN-028) and some have been withdrawn because of their low activity or adverse pharmacokinetic parameters. Some compounds are called “dual” inhibitors, allowing FLT3 inhibition associated with the inhibition of other kinases, such as AMG925 (CDK4/FLT3), SEL24-B489 (PIM/FLT3), CG806 (BTK/FLT3) or TAK-659 (SYK/FLT3). Gilteritinib is usually reported as a “dual” inhibitor (AXL/FLT3).

### 5.2. KIT Tyrosine Kinase Inhibitors

KIT (CD117) is the receptor for the SCF that is expressed on normal HSPC [15]. It is also expressed in 70% of AML and is mutated in 6%–8%, a rate increasing to 30%–46% in the subset of core-binding factor (CBF) AML [88,89,90,91]. This high frequency of mutated KIT in CBF AML suggests that its targeting could be one way to improve these AML outcomes, even if its role in normal hematopoiesis points to potential hematological toxicity and other side effects. Various mutations can affect KIT, the main ones being in exon 8 (extracellular domain 5) and 17 (intracellular TK domain). KIT mutation in exon 8 is a gain of function mutation (D419) inducing dimerization independently of its ligand, while mutations in exon 17 (D816V/N822) are the major mutation in the kinase domain. Their prognostic values are inconsistent [92,93,94]. In combination with chemotherapy, the KIT TKI dasatinib induces P53-dependent AML cell death [95]. In addition, both dasatinib and radotinib induced the death of AML cells by targeting KIT (Figure 2) [96]. SU5416 and SU6668 are multitargeted TKIs that inhibit VEGFR, KIT and FLT3. Their anti-angiogenic properties suggested a broader effect on AML cells expressing a high level of VEGF. While several studies confirmed their potential for inhibition, they seem to have too short a half-life [97,98].

### 5.3. TAM Tyrosine Kinase Inhibitors

The TAM receptor family includes AXL, TYRO3 and MER [99]. AXL is a class X RTK, which was cloned from CML cells [100]. It is activated by homodimerization upon the binding of its major ligand growth arrest-specific 6 (GAS6), while new ligands have been discovered for MER TK [101,102]. The GAS6/AXL pathway contributes to cell growth, survival, invasiveness, chemotaxis, apoptotic body clearance and immunity [103]. AXL is overexpressed in a wide variety of cancers [104]. In AML, high levels of expression of AXL and GAS6 have been related to poor outcomes [105,106] and in CML to resistance to BCR-ABL1 TKI [107,108,109]. Paracrine AXL activation has been shown to induce AML resistance to conventional chemotherapies and to FLT3-targeted therapy [110,111,112,113]. Recently, we reported a specific mechanism in the hematopoietic niche involving STAT5 and hypoxia, which mediates increased AXL expression and activation in AML cells [114]. The involvement of AXL in oncogenic cooperation in a wide range of malignancies made it the “perfect target” [102,115,116,117].

At least 19 AXL TKIs are in phase I/II or preclinical development for cancer therapies [102,118]. However, only bemcentinib (R428, BGB324) is currently being tested in AML. It has been developed by Rigel Pharmaceuticals and used in breast cancer and chronic lymphoid leukemia (CLL) [119,120]. It is now in a clinical trial in AML, but also in NSCLC (non-small-cell lung carcinoma) in combination with erlotinib [116,121,122,123]. Tyro-3 has been detected in several AML cell lines and its signaling is often deregulated [115,124]. ONO-9330547 has been developed as an AXL and MER TKI. This MER/AXL inhibitor has activity against FLT3-ITD AML cells by blocking the cell cycle through CDK/RB/PLK1 inhibition [125,126]. MER TK is also thought to be an interesting target in AML [127,128]. ONO-7475 was shown to have in vivo activity against both unmutated FLT3 and FLT3-ITD cell lines [129,130]. Cabozantinib is a multikinase inhibitor [131] that has been FDA approved in thyroid and renal carcinoma. Used in AML, it blocked FLT3-ITD and FLT3-TKD F691 mutants and was well tolerated, but it requires further investigation [132]. Recently, DS-1205, a new AXL inhibitor, was developed to overcome AXL-mediated resistance to EGFR-TKI in lung cancer. It may soon be transposed and tested in AML [133].

### 5.4. SYK Tyrosine Kinase Inhibitors

SYK TK is well known for its role in immune receptor signaling. Several studies have demonstrated its implication in AML through an increase in expression/phosphorylation, which was correlated to poor outcomes [50,51,134,135]. Despite the development of many SYK TKIs for immunological or lymphoproliferative diseases, their application in AML has been recently studied and reviewed [136].

Fostamatinib (R788) showed interesting STAT5 inhibition in preclinical studies in FLT3-ITD AML [137]. Entospletinib (GS-9973), a specific SYK TKI (IC50 = 7.6 nM), demonstrated significant single-agent activity, but a stronger effect in combination with chemotherapy in a phase I/II trial in AML [138]. Recent results of a phase II trial in t(11q23.3)/MLL AML and ALL patients reported at an AACR (American Association for Cancer Research) meeting showed a strong effect in monotherapy and a high response rate in combination with chemotherapy [139]. TAK-659 is a SYK/FLT3 TKI that is available for oral administration [140]. A phase 1b/2 clinical trial in R/R AML patients is ongoing (NCT02323113) [141]. Results from clinical trials in lymphoma are very encouraging and deserve further research in AML.

### 5.5. SFK Tyrosine Kinase Inhibitors

Several SRC family kinases (SFKs) are involved in AML (LYN, FYN, HCK, LCK and FGR). In normal hematopoiesis, transient expression and various roles of SFK have been reported such as the blockade of differentiation [142,143]. In AML, LYN is one of the predominant overexpressed SFKs and has been previously targeted using PD166285 [144]. However, different levels of expression are detected in chronic and acute leukemia [145]. In addition to overexpression, LYN is involved downstream of FLT3-ITD through direct interaction with FLT3 [146,147,148]. SFKs are reported to be activated downstream of FLT3-ITD but not FLT3-TKD [149,150]. Various SFKs can activate STAT5 [142] and the combination of SRC and FLT3 TKI is additive only in FLT3-ITD AML cells [151]. To date, dasatinib and ponatinib have been used in AML and have shown activity against FLT3-ITD AML [95,152]. Several clinical trials are ongoing using ponatinib in FLT3-ITD AML consolidation (NCT02428543), with or without azacytidine in FLT3-mutated AML (NCT02829840), or for preventing relapse after HSCT (NCT03690115). New TKIs are under development, such as SAR103168, a multikinase inhibitor inhibiting all the SFKs [153], the TL02-59, a dual inhibitor of FES/FLT3 and FGR TKI (TL02-59) [154,155].

### 5.6. MET/RON Tyrosine Kinase Inhibitors

The two RTKs in this family, MET and RON, have been detected in AML cells [156,157]. They belong to the class VIII RTK family and have a specific pattern of expression, which is overexpressed in a wide range of cancers and in various splicing forms. The MET ligand (HGF) exerts autocrine signaling in AML disrupted by crizotinib. However, resistance may occur and can be bypassed only by using combined therapies that involve inhibition of MET and FGFR [156,158]. MET TKI SU11274 blocked MEIS1/HOXA9-induced leukemia in an AML murine model, suggesting its potential [159]. Another TKI, BMS777607, has a wide range of targets, including TAM-R, RON and MET.

### 5.7. TEC Family Tyrosine Kinase Inhibitors

The TEC family is composed of five members (BMX, BTK, ITK, TEC and TXK) [160]. Bruton’s TK (BTK) was cloned in 1993 and its interaction with KIT was reported [161,162], leading to a mechanistic study in KIT-expressing AML. The BTK TKI ibrutinib, which is already used in CLL, showed that it is worthwhile targeting BTK in AML [163] and particularly in FLT3-ITD AML [164]. In addition, ibrutinib inhibits the SDF1/CXCR4-mediated migration of AML cells [165]. FDA approved for lymphoid malignancies, ibrutinib targeted FLT3-ITD AML cells in preclinical models and showed activity against some TKD mutants [166,167].

## 6. Concluding Remarks

For the last forty years, AML treatment has been limited to intensive chemotherapy with cytarabine and anthracycline. Deciphering the molecular landscape of AML has allowed accurate prognostic classification. One of the more mutated genes in AML is the RTK FLT3 with a frequency around 30%. This has given rise to the development of specific inhibitors to block the signaling of this RTK. The targeted therapies, including TKI, have improved outcomes in R/R AML and are currently being studied in combination with intensive chemotherapies or hypomethylating agents. In addition to the development of TKIs in AML, many other kinases or phosphatases can be targeted. For example, PTPN11, the SHP-2 phosphatase is a regulator downstream of many TKs that could be an additional target, like the similar SHP-1 (PTPN6), which is downregulated in CML and FLT3-ITD AML.

Onco-theranostic strategies are making a comeback in AML but will probably not be sufficient to obtain a definitive cure. Future associations with immune-oncologic strategies could be an interesting option to get the best out of these treatments.

## Figures and Tables

**Table 1:** TKI-based clinical trials in acute myeloid leukemia (recruiting, active, not recruiting or completed trials).

Drug, TK Targeted and Development Status in AML			NCT Number
Midostaurin	FLT3	FDA approved in newly diagnosed AML	-
Gilteritinib	FLT3	FDA approved in R/R AML	-
Quizartinib	FLT3	MHLW of Japan approved in R/R AML	-
Crenolanib	FLT3	7 phases 1 to 3 studies recruiting or active not recruiting in Clinical trials	-
Sorafenib	FLT3	15 phases 1 to 3 studies recruiting or active not recruiting in Clinical trials	-
Sunitinib	FLT3	Phase 1 and 1/2 recruiting and active	NCT01620216
Lestaurtinib	FLT3	Phase 1/2 completed study in R/R AML	NCT00469859
FF-10101	FLT3	Phase 1/2 recruiting study in R/R AML	NCT03194685
SEL24-B489	FLT3	Phase 1/2 recruiting study in newly diagnosed or R/R AML	NCT03008187
TAK-659	FLT3	Phase 1/2 completed study in newly diagnosed or R/R AML	NCT02323113
Dasatinib	KIT	13 phases 1 to 3 studies recruiting or active not recruiting in Clinical trials	-
SU5416	KIT	Phase 1/2 completed study in newly diagnosed or R/R AML	NCT00005942
Bemcentinib	AXL	Phase 2 recruiting study in newly diagnosed AML	NCT03824080
Cabozantinib	AXL	Phase 1 completed study in newly diagnosed or R/R AML	NCT01961765
Entospletinib	SYK	Phase 1/2 completed study in newly diagnosed or R/R AML	NCT02343939
SAR103168	SFK	Phase 1 completed study in R/R AML	NCT00981240
Ibrutinib	Btk	Phase 1 completed study in R/R AML	NCT02635074

## References

[1] Robinson D.R., Wu Y.M., Lin S.F. (2000). The protein tyrosine kinase family of the human genome. Oncogene.

[2] Manning G., Whyte D.B., Martinez R., Hunter T., Sudarsanam S. (2002). The protein kinase complement of the human genome. Science.

[3] Wang H., Chevalier D., Larue C., Ki Cho S., Walker J.C. (2007). The protein phosphatases and protein kinases of *Arabidopsis thaliana*. Arabidopsis Book.

[4] Eckhart W., Hutchinson M., Hunter T. (1979). An activity phosphorylating tyrosine in polyoma T antigen immunoprecipitates. Cell.

[5] Weiss A., Schlessinger J. (1998). Switching signals on or off by receptor dimerization. Cell.

[6] Duong-Ly K.C., Peterson J.R. (2013). The human kinome and kinase inhibition. Curr. Prot. Pharmacol..

[7] Traxler P.M., Furet P., Mett H., Buchdunger E., Meyer T., Lydon N. (1996). 4-(Phenylamino)pyrrolopyrimidines: Potent and selective, ATP site directed inhibitors of the EGF-receptor protein tyrosine kinase. J. Med. Chem..

[8] Carroll M. (1997). CGP 57148, a tyrosine kinase inhibitor, inhibits the growth of cells expressing BCR-ABL, TEL-ABL, and TEL-PDGFR fusion proteins. Blood.

[9] Fleuren E.D., Zhang L., Wu J., Daly R.J. (2016). The kinome ‘at large’ in cancer. Nat. Rev. Cancer.

[10] Gross S., Rahal R., Stransky N., Lengauer C., Hoeflich K.P. (2015). Targeting cancer with kinase inhibitors. J. Clin. Invest..

[11] Roskoski R. (2016). Classification of small molecule protein kinase inhibitors based upon the structures of their drug-enzyme complexes. Pharmacol. Res..

[12] Bhullar K.S., Lagaron N.O., McGowan E.M., Parmar I., Jha A., Hubbard B.P., Rupasinghe H.P.V. (2018). Kinase-targeted cancer therapies: Progress, challenges and future directions. Mol. Cancer.

[13] Paulson R.F., Bernstein A. (1995). Receptor tyrosine kinases and the regulation of hematopoiesis. Semin. Immunol..

[14] Rohrschneider L.R., Bourette R.P., Lioubin M.N., Algate P.A., Myles G.M., Carlberg K. (1997). Growth and differentiation signals regulated by the M-CSF receptor. Mol. Reprod. Dev..

[15] Bernstein A., Forrester L., Reith A.D., Dubreuil P., Rottapel R. (1991). The murine W/c-kit and Steel loci and the control of hematopoiesis. Semin. Hematol..

[16] Gilliland D.G., Griffin J.D. (2002). Role of FLT3 in leukemia. Curr. Opin. Hematol..

[17] Ihle J.N. (1995). TheJanusprotein tyrosine kinases in hematopoietic cytokine signaling. Semin. Immonol..

[18] Harrison D.A., Binari R., Nahreini T.S., Gilman M., Perrimon N. (1995). Activation of a Drosophila Janus kinase (JAK) causes hematopoietic neoplasia and developmental defects. EMBO J..

[19] Chow L.M.L., Veillette A. (1995). The Src and Csk families of tyrosine protein kinases in hemopoietic cells. Semin. Immonol..

[20] Corey S.J., Anderson S.M. (1999). Src-related protein tyrosine kinases in hematopoiesis. Blood.

[21] Ingley E. (2008). Src family kinases: Regulation of their activities, levels and identification of new pathways. Biochim. Biophys. Acta (BBA) Proteins Proteom..

[22] Klingmüller U., Wu H., Hsiao J.G., Toker A., Duckworth B.C., Cantley L.C., Lodish H.F. (1997). Identification of a novel pathway important for proliferation and differentiation of primary erythroid progenitors. Proc. Natl. Acad. Sci. USA.

[23] Lannutti B.J., Shim M.-H., Blake N., Reems J.A., Drachman J.G. (2003). Identification and activation of Src family kinases in primary megakaryocytes. Exp. Hematol..

[24] Hibbs M.L., Tarlinton D.M., Armes J., Grail D., Hodgson G., Maglitto R., Stacker S.A., Dunn A.R. (1995). Multiple defects in the immune system of Lyn-deficient mice, culminating in autoimmune disease. Cell.

[25] Stein P.L., Vogel H., Soriano P. (1994). Combined deficiencies of Src, Fyn, and Yes tyrosine kinases in mutant mice. Genes Dev..

[26] Lowell C.A., Niwa M., Soriano P., Varmus H.E. (1996). Deficiency of the Hck and Src tyrosine kinases results in extreme levels of extramedullary hematopoiesis. Blood.

[27] Roche S., Koegl M., Barone M.V., Roussel M.F., Courtneidge S.A. (1995). DNA synthesis induced by some but not all growth factors requires Src family protein tyrosine kinases. Mol. Cell Biol..

[28] Corey S.J., Dombrosky-Ferlan P.M., Zuo S., Krohn E., Donnenberg A.D., Zorich P., Romero G., Takata M., Kurosaki T. (1998). Requirement of src kinase lyn for induction of DNA synthesis by granulocyte colony-stimulating factor. J. Biol. Chem..

[29] Linnekin D., DeBerry C.S., Mou S. (1997). Lyn associates with the juxtamembrane region of c-Kit and is activated by stem cell factor in hematopoietic cell lines and normal progenitor cells. J. Biol. Chem..

[30] Mano H., Ishikawa F., Nishida J., Hirai H., Takaku F. (1990). A novel protein-tyrosine kinase, tec, is preferentially expressed in liver. Oncogene.

[31] Yang W.-C., Collette Y., Nunes J.A., Olive D. (2000). Tec kinases: A family with multiple roles in immunity. Immunity.

[32] Vihinen M., Brandau O., Brandén L.J., Kwan S.P., Lappalainen I., Lester T., Noordzij J.G., Ochs H.D., Ollila J., Pienaar S.M. (1998). BTKbase, mutation database for X-linked agammaglobulinemia (XLA). Nucleic Acids Res..

[33] Van Oers N.S.C., Weiss A. (1995). The Syk/ZAP-70 protein tyrosine kinase connection to antigen receptor signalling processes. Semin. Immonol..

[34] Latour S., Chow L.M.L., Veillette A. (1996). Differential intrinsic enzymatic activity of Syk and Zap-70 protein-tyrosine kinases. J. Biol. Chem..

[35] Efremov D.G., Laurenti L. (2011). The Syk kinase as a therapeutic target in leukemia and lymphoma. Expert Opin. Invest. Drugs.

[36] Cheng A.M., Rowley B., Pao W., Hayday A., Bolen J.B., Pawson T. (1995). Syk tyrosine kinase required for mouse viability and B-cell development. Nature.

[37] Turner M., Joseph Mee P., Costello P.S., Williams O., Price A.A., Duddy L.P., Furlong M.T., Geahlen R.L., Tybulewicz V.L.J. (1995). Perinatal lethality and blocked B-cell development in mice lacking the tyrosine kinase Syk. Nature.

[38] MacDonald I., Levy J., Pawson T. (1985). Expression of the mammalian c-fes protein in hematopoietic cells and identification of a distinct fes-related protein. Mol. Cell Biol..

[39] Yates K.E., Gasson J.C. (1996). Role of c-Fes in normal and neoplastic hematopoiesis. Stem Cell J..

[40] Craig A.W. (2012). FES/FER kinase signaling in hematopoietic cells and leukemias. Front Biosci..

[41] Blume-Jensen P., Hunter T. (2001). Oncogenic kinase signalling. Nature.

[42] Scheijen B., Griffin J.D. (2002). Tyrosine kinase oncogenes in normal hematopoiesis and hematological disease. Oncogene.

[43] Ku M., Wall M., MacKinnon R.N., Walkley C.R., Purton L.E., Tam C., Izon D., Campbell L., Cheng H.-C., Nandurkar H. (2015). Src family kinases and their role in hematological malignancies. Leuk. Lymphoma.

[44] Morris C., Kennedy M., Heisterkamp N., Columbano-Green L., Romeril K., Groffen J., Fitzgerald P. (1991). A complex chromosome rearrangement forms the BCR-ABL fusion gene in leukemic cells with a normal karyotype. Genes Chromosomes Cancer.

[45] Patel J.P., Gonen M., Figueroa M.E., Fernandez H., Sun Z., Racevskis J., Van Vlierberghe P., Dolgalev I., Thomas S., Aminova O. (2012). Prognostic relevance of integrated genetic profiling in acute myeloid leukemia. N. Engl. J. Med..

[46] Kaushansky K. (2005). On the molecular origins of the chronic myeloproliferative disorders: It all makes sense. Blood.

[47] James C., Ugo V., Le Couedic J.P., Staerk J., Delhommeau F., Lacout C., Garcon L., Raslova H., Berger R., Bennaceur-Griscelli A. (2005). A unique clonal JAK2 mutation leading to constitutive signalling causes polycythaemia vera. Nature.

[48] Deschler B., Lübbert M. (2006). Acute myeloid leukemia: Epidemiology and etiology. Int. J. Am. Cancer Soc..

[49] Bullinger L., Döhner K., Döhner H. (2017). Genomics of acute myeloid leukemia diagnosis and pathways. J. Clin. Oncol..

[50] Hahn C.K., Berchuck J.E., Ross K.N., Kakoza R.M., Clauser K., Schinzel A.C., Ross L., Galinsky I., Davis T.N., Silver S.J. (2009). Proteomic and genetic approaches identify Syk as an AML target. Cancer Cell.

[51] Puissant A., Fenouille N., Alexe G., Pikman Y., Bassil C.F., Mehta S., Du J., Kazi J.U., Luciano F., Rönnstrand L. (2014). SYK is a critical regulator of FLT3 in acute myeloid leukemia. Cancer Cell.

[52] Farge T., Saland E., de Toni F., Aroua N., Hosseini M., Perry R., Bosc C., Sugita M., Stuani L., Fraisse M. (2017). Chemotherapy-resistant human acute myeloid leukemia cells are not enriched for leukemic stem cells but require oxidative metabolism. Cancer Discov..

[53] Kainz B., Heintel D., Marculescu R., Schwarzinger I., Sperr W., Le T., Weltermann A., Fonatsch C., Haas O.A., Mannhalter C. (2002). Variable prognostic value of FLT3 internal tandem duplications in patients with de novo AML and a normal karyotype, t(15;17), t(8;21) or inv(16). Hematol. J..

[54] Stirewalt D.L., Radich J.P. (2003). The role of FLT3 in haematopoietic malignancies. Nat. Rev. Cancer.

[55] Toffalini F., Demoulin J.-B. (2010). New insights into the mechanisms of hematopoietic cell transformation by activated receptor tyrosine kinases. Blood.

[56] Whitman S.P., Maharry K., Radmacher M.D., Becker H., Mrozek K., Margeson D., Holland K.B., Wu Y.Z., Schwind S., Metzeler K.H. (2010). FLT3 internal tandem duplication associates with adverse outcome and gene- and microRNA-expression signatures in patients 60 years of age or older with primary cytogenetically normal acute myeloid leukemia: A cancer and leukemia group B study. Blood.

[57] Sharma M., Ravandi F., Bayraktar U.D., Chiattone A., Bashir Q., Giralt S., Chen J., Qazilbash M., Kebriaei P., Konopleva M. (2011). Treatment of FLT3-ITD-positive acute myeloid leukemia relapsing after allogeneic stem cell transplantation with Sorafenib. Biol. Blood Marrow Transplant..

[58] Kindler T., Lipka D.B., Fischer T. (2010). FLT3 as a therapeutic target in AML: Still challenging after all these years. Blood.

[59] Grunwald M.R., Levis M.J. (2013). FLT3 inhibitors for acute myeloid leukemia: A review of their efficacy and mechanisms of resistance. Int. J. Hematol..

[60] Levis M., Allebach J., Tse K.F., Zheng R., Baldwin B.R., Smith B.D., Jones-Bolin S., Ruggeri B., Dionne C., Small D. (2002). A FLT3-targeted tyrosine kinase inhibitor is cytotoxic to leukemia cells in vitro and in vivo. Blood.

[61] Levis M., Ravandi F., Wang E.S., Baer M.R., Perl A., Coutre S., Erba H., Stuart R.K., Baccarani M., Cripe L.D. (2011). Results from a randomized trial of salvage chemotherapy followed by lestaurtinib for patients with FLT3 mutant AML in first relapse. Blood.

[62] Rahmani M., Davis E.M., Bauer C., Dent P., Grant S. (2005). Apoptosis induced by the kinase inhibitor BAY 43-9006 in human leukemia cells involves down-regulation of Mcl-1 through inhibition of translation. J. Biol. Chem..

[63] Zhang W., Konopleva M., Ruvolo V.R., McQueen T., Evans R.L., Bornmann W.G., McCubrey J., Cortes J., Andreeff M. (2008). Sorafenib induces apoptosis of AML cells via Bim-mediated activation of the intrinsic apoptotic pathway. Leukemia.

[64] Burchert A., Bug G., Finke J., Stelljes M., Rollig C., Wäsch R., Bornhauser M., Berg T., Lang F., Ehninger G. (2018). Sorafenib as maintenance therapy post allogeneic stem cell transplantation for FLT3-ITD positive AML: Results from the randomized, double-blind, placebo-controlled multicentre sormain trial. Blood.

[65] Mathew N.R., Baumgartner F., Braun L., O’Sullivan D., Thomas S., Waterhouse M., Muller T.A., Hanke K., Taromi S., Apostolova P. (2018). Sorafenib promotes graft-versus-leukemia activity in mice and humans through IL-15 production in FLT3-ITD-mutant leukemia cells. Nat. Med..

[66] Weisberg E., Boulton C., Kelly L.M., Manley P., Fabbro D., Meyer T., Gilliland D.G., Griffin J.D. (2002). Inhibition of mutant FLT3 receptors in leukemia cells by the small molecule tyrosine kinase inhibitor PKC412. Cancer Cell.

[67] Stone R.M., Manley P.W., Larson R.A., Capdeville R. (2018). Midostaurin: Its odyssey from discovery to approval for treating acute myeloid leukemia and advanced systemic mastocytosis. Blood Adv..

[68] O’Farrell A.-M., Abrams T.J., Yuen H.A., Ngai T.J., Louie S.G., Yee K.W.H., Wong L.M., Hong W., Lee L.B., Town A. (2003). SU11248 is a novel FLT3 tyrosine kinase inhibitor with potent activity in vitro and in vivo. Blood.

[69] Fiedler W., Serve H., Dohner H., Schwittay M., Ottmann O.G., O’Farrell A.-M., Bello C.L., Allred R., Manning W.C., Cherrington J.M. (2005). A phase 1 study of SU11248 in the treatment of patients with refractory or resistant acute myeloid leukemia (AML) or not amenable to conventional therapy for the disease. Blood.

[70] Fiedler W., Kayser S., Kebenko M., Janning M., Krauter J., Schittenhelm M., Gotze K., Weber D., Gohring G., Teleanu V. (2015). A phase I/II study of sunitinib and intensive chemotherapy in patients over 60 years of age with acute myeloid leukaemia and activating FLT3 mutations. Br. J. Haematol..

[71] Zarrinkar P.P., Gunawardane R.N., Cramer M.D., Gardner M.F., Brigham D., Belli B., Karaman M.W., Pratz K.W., Pallares G., Chao Q. (2009). AC220 is a uniquely potent and selective inhibitor of FLT3 for the treatment of acute myeloid leukemia (AML). Blood.

[72] Levis M. (2014). Quizartinib for the treatment of FLT3/ITD acute myeloid leukemia. Future Oncol..

[73] Altman J.K., Foran J.M., Pratz K.W., Trone D., Cortes J.E., Tallman M.S. (2013). Phase 1 study of quizartinib in combination with induction and consolidation chemotherapy in patients with newly diagnosed acute myeloid leukemia. Am. J. Hematol..

[74] Hills R.K., Gammon G., Trone D., Burnett A.K. (2015). Quizartinib significantly improves overall survival in FLT3-ITD positive AML patients relapsed after stem cell transplantation or after failure of salvage chemotherapy: A comparison with historical AML database (UK NCRI data). Blood.

[75] Cortes J., Perl A.E., Dohner H., Kantarjian H., Martinelli G., Kovacsovics T., Rousselot P., Steffen B., Dombret H., Estey E. (2018). Quizartinib, an FLT3 inhibitor, as monotherapy in patients with relapsed or refractory acute myeloid leukaemia: An open-label, multicentre, single-arm, phase 2 trial. Lancet.

[76] Cortes J.E., Khaled S.K., Martinelli G., Perl A.E., Ganguly S., Russell N.H., Kramer A., Dombret H., Hogge D., Jonas B.A. (2018). Efficacy and safety of single-agent Quizartinib (Q), a potent and selective FLT3 inhibitor (FLT3i), in patients (pts) with FLT3-internal tandem duplication (FLT3-ITD)-mutated relapsed/refractory (R/R) acute myeloid leukemia (AML) enrolled in the global, phase 3, randomized controlled quantum-R trial. Blood.

[77] Zimmerman E.I., Turner D.C., Buaboonnam J., Hu S., Orwick S., Roberts M.S., Janke L.J., Ramachandran A., Stewart C.F., Inaba H. (2013). Crenolanib is active against models of drug-resistant FLT3-ITD−positive acute myeloid leukemia. Blood.

[78] Galanis A., Ma H., Rajkhowa T., Ramachandran A., Small D., Cortes J., Levis M. (2014). Crenolanib is a potent inhibitor of FLT3 with activity against resistance-conferring point mutants. Blood.

[79] Randhawa J.K., Kantarjian H.M., Borthakur G., Thompson P.A., Konopleva M., Daver N., Pemmaraju N., Jabbour E., Kadia T.M., Estrov Z. (2014). Results of a phase II study of crenolanib in relapsed/refractory acute myeloid leukemia patients (Pts) with activating FLT3 mutations. Blood.

[80] Jorge E.C., Hagop M.K., Tapan M.K., Gautam B., Marina K., Guillermo G.-M., Naval Guastad D., Naveen P., Elias J., Zeev E. (2016). Crenolanib besylate, a type I pan-FLT3 inhibitor, to demonstrate clinical activity in multiply relapsed FLT3-ITD and D835 AML. J. Clin. Oncol..

[81] Zhang H., Savage S., Schultz A.R., Bottomly D., White L., Segerdell E., Wilmot B., McWeeney S.K., Eide C.A., Nechiporuk T. (2019). Clinical resistance to crenolanib in acute myeloid leukemia due to diverse molecular mechanisms. Nat. Commun..

[82] Levis M., Alexander E.P., Jessica K.A., Jorge E.C., Ellen K.R., Richard A.L., Catherine Choy S., Eunice S.W., Stephen Anthony S., Maria R.B. (2015). Results of a first-in-human, phase I/II trial of ASP2215, a selective, potent inhibitor of FLT3/Axl in patients with relapsed or refractory (R/R) acute myeloid leukemia (AML). J. Clin. Oncol..

[83] Perl A.E., Altman J.K., Cortes J., Smith C., Litzow M., Baer M.R., Claxton D., Erba H.P., Gill S., Goldberg S. (2017). Selective inhibition of FLT3 by gilteritinib in relapsed or refractory acute myeloid leukaemia: A multicentre, first-in-human, open-label, phase 1–2 study. Lancet Oncol..

[84] McMahon C.M., Ferng T., Canaani J., Wang E.S., Morrissette J.J.D., Eastburn D.J., Pellegrino M., Durruthy-Durruthy R., Watt C.D., Asthana S. (2019). Clonal selection with Ras pathway activation mediates secondary clinical resistance to selective FLT3 inhibition in acute myeloid leukemia. Cancer Discov..

[85] Perl A., Martinelli G., Cortes J. Gilteritinib significantly prolongs overall survival in patients with FLT3-mutated (FLT3mut+) relapsed/refractory (R/R) acute myeloid leukemia (AML): Results from the phase III ADMIRAL trial. Proceedings of the AACR Annual Meeting.

[86] Nakatani T., Uda K., Yamaura T., Takasaki M., Akashi A., Chen F., Ishikawa Y., Hayakawa F., Hagiwara S., Kiyoi H. (2015). Development of FF-10101, a novel irreversible FLT3 inhibitor, which overcomes drug resistance mutations. Blood.

[87] Yamaura T., Nakatani T., Uda K., Ogura H., Shin W., Kurokawa N., Saito K., Fujikawa N., Date T., Takasaki M. (2018). A novel irreversible FLT3 inhibitor, FF-10101, shows excellent efficacy against AML cells with FLT3 mutations. Blood.

[88] Ikeda H., Kanakura Y., Tamaki T., Kuriu A., Kitayama H., Ishikawa J., Kanayama Y., Yonezawa T., Tarui S., Griffin J.D. (1991). Expression and functional role of the proto-oncogene c-kit in acute myeloblastic leukemia cells. Blood.

[89] Cairoli R., Beghini A., Grillo G., Nadali G., Elice F., Ripamonti C.B., Colapietro P., Nichelatti M., Pezzetti L., Lunghi M. (2006). Prognostic impact of c-KIT mutations in core binding factor leukemias: An Italian retrospective study. Blood.

[90] Malaise M., Steinbach D., Corbacioglu S. (2009). Clinical implications of c-Kit mutations in acute myelogenous leukemia. Curr. Hematol. Malig. Rep..

[91] Ashman L.K., Griffith R. (2013). Therapeutic targeting of c-KIT in cancer. Expert Opin. Invest. Drugs.

[92] Lennartsson J., Jelacic T., Linnekin D., Shivakrupa R. (2005). Normal and oncogenic forms of the receptor tyrosine kinase kit. Stem Cells.

[93] Chen W., Xie H., Wang H., Chen L., Sun Y., Chen Z., Li Q. (2016). Prognostic significance of KIT mutations in core-binding factor acute myeloid leukemia: A systematic review and meta-analysis. PLoS ONE.

[94] Ayatollahi H., Shajiei A., Sadeghian M.H., Sheikhi M., Yazdandoust E., Ghazanfarpour M., Shams S.F., Shakeri S. (2017). Prognostic importance of C-KIT mutations in core binding factor acute myeloid leukemia: A systematic review. Hematol. Oncol. Stem Cell Ther..

[95] Dos Santos C., McDonald T., Ho Y.W., Liu H., Lin A., Forman S.J., Kuo Y.-H., Bhatia R. (2013). The Src and c-Kit kinase inhibitor dasatinib enhances p53-mediated targeting of human acute myeloid leukemia stem cells by chemotherapeutic agents. Blood.

[96] Heo S.-K., Noh E.-K., Kim J.Y., Jeong Y.K., Jo J.-C., Choi Y., Koh S., Baek J.H., Min Y.J., Kim H. (2017). Targeting c-KIT (CD117) by dasatinib and radotinib promotes acute myeloid leukemia cell death. Sci. Rep..

[97] Fiedler W., Mesters R., Tinnefeld H., Loges S., Staib P., Dührsen U., Flasshove M., Ottmann O.G., Jung W., Cavalli F. (2003). A phase 2 clinical study of SU5416 in patients with refractory acute myeloid leukemia. Blood.

[98] Smolich B.D., Yuen H.A., West K.A., Giles F.J., Albitar M., Cherrington J.M. (2001). The antiangiogenic protein kinase inhibitors SU5416 and SU6668 inhibit the SCF receptor (c-kit) in a human myeloid leukemia cell line and in acute myeloid leukemia blasts. Blood.

[99] Linger R.M.A., Keating A.K., Earp H.S., Graham D.K. (2008). TAM receptor tyrosine kinases: Biologic functions, signaling, and potential therapeutic targeting in human cancer. Adv. Cancer Res..

[100] Neubauer A., O’Bryan J.P., Fiebeler A., Schmidt C., Huhn D., Liu E.T. (1993). Axl, a novel receptor tyrosine kinase isolated from chronic myelogenous leukemia. Semin. Hematol..

[101] Caberoy N.B., Zhou Y., Li W. (2010). Tubby and tubby-like protein 1 are new MerTK ligands for phagocytosis. EMBO J..

[102] Schoumacher M., Burbridge M. (2017). Key roles of AXL and MER receptor tyrosine kinases in resistance to multiple anticancer therapies. Curr. Oncol. Rep..

[103] Graham D.K., DeRyckere D., Davies K.D., Earp H.S. (2014). The TAM family: Phosphatidylserine-sensing receptor tyrosine kinases gone awry in cancer. Nat. Rev. Cancer.

[104] Mollard A., Warner S.L., Call L.T., Wade M.L., Bearss J.J., Verma A., Sharma S., Vankayalapati H., Bearss D.J. (2011). Design, synthesis and biological evaluation of a series of novel Axl kinase inhibitors. ACS Med. Chem. Lett..

[105] Rochlitz C., Lohri A., Bacchi M., Schmidt M., Nagel S., Fopp M., Fey M.F., Herrmann R., Neubauer A. (1999). Axl expression is associated with adverse prognosis and with expression of Bcl-2 and CD34 in de novo acute myeloid leukemia (AML): Results from a multicenter trial of the Swiss group for clinical cancer research (SAKK). Leukemia.

[106] Whitman S.P., Kohlschmidt J., Maharry K., Volinia S., Mrozek K., Nicolet D., Schwind S., Becker H., Metzeler K.H., Mendler J.H. (2014). GAS6 expression identifies high-risk adult AML patients: Potential implications for therapy. Leukemia.

[107] Dufies M., Jacquel A., Belhacene N., Robert G., Cluzeau T., Luciano F., Cassuto J.P., Raynaud S., Auberger P. (2011). Mechanisms of AXL overexpression and function in Imatinib-resistant chronic myeloid leukemia cells. Oncotarget.

[108] Gioia R., Leroy C., Drullion C., Lagarde V., Etienne G., Dulucq S., Lippert E., Roche S., Mahon F.X., Pasquet J.M. (2011). Quantitative phosphoproteomics revealed interplay between Syk and Lyn in the resistance to nilotinib in chronic myeloid leukemia cells. Blood.

[109] Gioia R., Tregoat C., Dumas P.Y., Lagarde V., Prouzet-Mauleon V., Desplat V., Sirvent A., Praloran V., Lippert E., Villacreces A. (2015). CBL controls a tyrosine kinase network involving AXL, SYK and LYN in nilotinib-resistant chronic myeloid leukaemia. J. Pathol..

[110] Hong C.C., Lay J.D., Huang J.S., Cheng A.L., Tang J.L., Lin M.T., Lai G.M., Chuang S.E. (2008). Receptor tyrosine kinase AXL is induced by chemotherapy drugs and overexpression of AXL confers drug resistance in acute myeloid leukemia. Cancer Lett..

[111] Ben-Batalla I., Schultze A., Wroblewski M., Erdmann R., Heuser M., Waizenegger J.S., Riecken K., Binder M., Schewe D., Sawall S. (2013). Axl, a prognostic and therapeutic target in acute myeloid leukemia mediates paracrine cross-talk of leukemia cells with bone marrow stroma. Blood.

[112] Park I.-K., Mishra A., Chandler J., Whitman S.P., Marcucci G., Caligiuri M.A. (2013). Inhibition of the receptor tyrosine kinase Axl impedes activation of the FLT3 internal tandem duplication in human acute myeloid leukemia: Implications for Axl as a potential therapeutic target. Blood.

[113] Park I.K., Mundy-Bosse B., Whitman S.P., Zhang X., Warner S.L., Bearss D.J., Blum W., Marcucci G., Caligiuri M.A. (2015). Receptor tyrosine kinase Axl is required for resistance of leukemic cells to FLT3-targeted therapy in acute myeloid leukemia. Leukemia.

[114] Dumas P.-Y., Naudin C.c., Martin-Lanner2e S.V., Izac B., Casetti L., Mansier O., Rousseau B.T., Artus A., Dufossée M.L., Giese A. (2019). Hematopoietic niche drives FLT3-ITD acute myeloid leukemia resistance to quizartinib via STAT5- and hypoxia- dependent up-regulation of AXL. Haematologica.

[115] Huey M.G., Minson K.A., Earp H.S., DeRyckere D., Graham D.K. (2016). Targeting the TAM receptors in leukemia. Cancers.

[116] Gay C.M., Balaji K., Byers L.A. (2017). Giving AXL the axe: Targeting AXL in human malignancy. Br. J. Cancer.

[117] Shen Y., Chen X., He J., Liao D., Zu X. (2018). Axl inhibitors as novel cancer therapeutic agents. Life Sci..

[118] Myers S.H., Brunton V.G., Unciti-Broceta A. (2016). AXL Inhibitors in cancer: A medicinal chemistry perspective. J. Med. Chem..

[119] Holland S.J., Pan A., Franci C., Hu Y., Chang B., Li W., Duan M., Torneros A., Yu J., Heckrodt T.J. (2010). R428, a selective small molecule inhibitor of Axl kinase, blocks tumor spread and prolongs survival in models of metastatic breast cancer. Cancer Res..

[120] Ghosh A.K., Secreto C., Boysen J., Sassoon T., Shanafelt T.D., Mukhopadhyay D., Kay N.E. (2010). A novel receptor tyrosine kinase Axl is constitutively active in B-cell chronic lymphocytic leukemia and acts as a docking site of non-receptor kinases: Implications for therapy. Blood.

[121] Loges S., Björn Tore G., Michael H., Jörg C., Carlos Enrique V., Peter P., Ben-Batalla I., Nuray A., David M., Anthony B. (2018). The immunomodulatory activity of bemcentinib (BGB324): A first-in-class selective oral AXL inhibitor in patients with relapsed/refractory acute myeloid leukemia or myelodysplastic syndrome. J. Clin. Oncol..

[122] Loges S., Bjorn Torre G., Michael H., Ben-Batalla I., David M., Chromik J., Maxim K., Walter M.F., Jorge E.C. (2016). A first-in-patient phase I study of BGB324, a selective Axl kinase inhibitor in patients with refractory/relapsed AML and high-risk MDS. J. Clin. Oncol..

[123] Sonja L., Gjertsen B.T., Heuser M., Chromik J., Batalla I.B., Akyüz N., Micklem D., Brown A., Lorens J., Kebenko M. (2016). BGB324, an orally available selective Axl inhibitor exerts anti-leukemic activity in the first-in-patient trial BGBC003 and induces unique changes in biomarker profiles. Blood.

[124] Eryildiz F., Tyner J.W. (2016). Abstract 1265: Dysregulated tyrosine kinase Tyro3 signaling in acute myeloid leukemia. Cancer Res..

[125] Gilmour M., Scholtz A., Ottmann O.G., Hills R.K., Knapper S., Zabkiewicz J. (2016). Axl/Mer Inhibitor ONO-9330547 As a novel therapeutic agent in a stromal co-culture model of primary acute myeloid leukaemia (AML). Blood.

[126] Ruvolo P., Ma H., Ruvolo V., Mu H., Schober W., Yasuhiro T., Yoshizawa T., Gallardo M., Zhang X., Khoury J.D. (2016). AXL inhibitor ONO-9330547 suppresses PLK1 gene and protein expression and effectively induces growth arrest and apoptosis in FLT3 ITD acute myeloid leukemia cells. Blood.

[127] Lee-Sherick A.B., Eisenman K.M., Sather S., McGranahan A., Armistead P.M., McGary C.S., Hunsucker S.A., Schlegel J., Martinson H., Cannon C. (2013). Aberrant Mer receptor tyrosine kinase expression contributes to leukemogenesis in acute myeloid leukemia. Oncogene.

[128] Minson K.A., Huey M.G., Hill A.A., Perez I., Wang X., Frye S., Earp H.S., DeRyckere D., Graham D.K. (2016). Bone marrow stromal cell mediated resistance to mertk inhibition in acute leukemia. Blood.

[129] Ruvolo P.P., Ma H., Ruvolo V.R., Zhang X., Mu H., Schober W., Hernandez I., Gallardo M., Khoury J.D., Cortes J. (2017). Anexelekto/MER tyrosine kinase inhibitor ONO-7475 arrests growth and kills FMS-like tyrosine kinase 3-internal tandem duplication mutant acute myeloid leukemia cells by diverse mechanisms. Haematologica.

[130] Tanaka K., Li C., Hirosaki T., Kato H., Ishikawa Y., Oka M., Egawa H., Kozaki R., Yoshizawa T. (2018). Abstract 1883: A novel Axl and Mertk dual inhibitor ONO-7475: A new therapeutic agent for the treatment of FLT3-ITD and -wild-type acute myeloid leukemia (AML) overexpressing. Cancer Res..

[131] Lu J.W., Wang A.N., Liao H.A., Chen C.Y., Hou H.A., Hu C.Y., Tien H.F., Ou D.L., Lin L.I. (2016). Cabozantinib is selectively cytotoxic in acute myeloid leukemia cells with FLT3-internal tandem duplication (FLT3-ITD). Cancer Lett..

[132] Fathi A.T., Blonquist T.M., Hernandez D., Amrein P.C., Ballen K.K., McMasters M., Avigan D.E., Joyce R., Logan E.K., Hobbs G. (2018). Cabozantinib is well tolerated in acute myeloid leukemia and effectively inhibits the resistance-conferring FLT3/tyrosine kinase domain/F691 mutation. Cancer.

[133] Jimbo T., Taira T., Komatsu T., Kumazawa K., Maeda N., Haginoya N., Suzuki T., Ota M., Totoki Y., Wada C. (2017). DS-1205b, a novel, selective, small-molecule inhibitor of AXL, delays the onset of resistance and overcomes acquired resistance to EGFR-TKIs in a human EGFR-mutant NSCLC (T790M-negative) xenograft model. Ann. Oncol..

[134] Oellerich T., Oellerich M.F., Engelke M., Munch S., Mohr S., Nimz M., Hsiao H.-H., Corso J., Zhang J., Bohnenberger H. (2013). b2 integrine derived signals induce cell survival and proliferation of AML blasts by activating a Syk/STAT signaling axis. Blood.

[135] Boros K., Puissant A., Back M., Alexe G., Bassil C.F., Sinha P., Tholouli E., Stegmaier K., Byers R.J., Rodig S.J. (2015). Increased SYK activity is associated with unfavorable outcome among patients with acute myeloid leukemia. Oncotarget.

[136] Bartaula-Brevik S., Lindstad Brattas M.K., Tvedt T.H.A., Reikvam H., Bruserud O. (2018). Splenic tyrosine kinase (SYK) inhibitors and their possible use in acute myeloid leukemia. Expert Opin. Invest. Drugs.

[137] Richine B.M., Virts E.L., Bowling J.D., Ramdas B., Mali R., Naoye R., Liu Z., Zhang Z.Y., Boswell H.S., Kapur R. (2016). Syk kinase and Shp2 phosphatase inhibition cooperate to reduce FLT3-ITD-induced STAT5 activation and proliferation of acute myeloid leukemia. Leukemia.

[138] Walker A.R., Bhatnagar B., Marcondes A.M.Q., DiPaolo J., Vasu S., Mims A.S., Klisovic R.B., Walsh K.J., Canning R., Behbehani G.K. (2016). Interim results of a phase 1b/2 study of entospletinib (GS-9973) monotherapy and in combination with chemotherapy in patients with acute myeloid leukemia. Blood.

[139] Walker A.R., Byrd J.C., Blum W., Lin T., Crosswell H.E., Zhang D., Gao J., Rao A.V., Minden M.D., Stock W. (2018). Abstract 819: High response rates with entospletinib in patients with t(v;11q23.3)KMT2A rearranged acute myeloid leukemia and acute lymphoblastic leukemia. Cancer Res.

[140] Jie Y., Jessica H., Matthew T., Helen H., Stephen T., Mengkun Z., Karuppiah K. (2016). Anti-tumor activity of TAK-659, a dual inhibitor of SYK and FLT-3 kinases, in AML models. J. Clin. Oncol..

[141] Kaplan J.B., Bixby D.L., Morris J.C., Frankfurt O., Altman J., Wise-Draper T., Burke P.W., Collins S., Kannan K., Wang L. (2016). A phase 1b/2 study of TAK-659, an investigational dual SYK and FLT-3 inhibitor, in patients (Pts) with relapsed or refractory acute myelogenous leukemia (R/R AML). Blood.

[142] Ozawa Y., Williams A.H., Estes M.L., Matsushita N., Boschelli F., Jove R., List A.F. (2008). Src family kinases promote AML cell survival through activation of signal transducers and activators of transcription (STAT). Leuk. Res..

[143] Marhall A., Kazi J.U., Ronnstrand L. (2017). The Src family kinase LCK cooperates with oncogenic FLT3/ITD in cellular transformation. Sci. Rep..

[144] Roginskaya V., Zuo S., Caudell E., Nambudiri G., Kraker A.J., Corey S.J. (1999). Therapeutic targeting of Src-kinase Lyn in myeloid leukemic cell growth. Leukemia.

[145] Hussein K., von Neuhoff N., Büsche G., Buhr T., Kreipe H., Bock O. (2009). Opposite expression pattern of Src kinase Lyn in acute and chronic haematological malignancies. Ann. Hematol..

[146] Robinson L.J., Xue J., Corey S.J. (2005). Src family tyrosine kinases are activated by Flt3 and are involved in the proliferative effects of leukemia-associated Flt3 mutations. Exp. Hematol..

[147] Dos Santos C., Demur C., Bardet V., Prade-Houdellier N., Payrastre B., Recher C. (2008). A critical role for Lyn in acute myeloid leukemia. Blood.

[148] Okamoto M., Hayakawa F., Miyata Y., Watamoto K., Emi N., Abe A., Kiyoi H., Towatari M., Naoe T. (2007). Lyn is an important component of the signal transduction pathway specific to FLT3/ITD and can be a therapeutic target in the treatment of AML with FLT3/ITD. Leukemia.

[149] Leischner H., Albers C., Grundler R., Razumovskaya E., Spiekermann K., Bohlander S., Ronnstrand L., Gotze K., Peschel C., Duyster J. (2012). SRC is a signaling mediator in FLT3-ITD- but not in FLT3-TKD-positive AML. Blood.

[150] Ingley E. (2012). Functions of the Lyn tyrosine kinase in health and disease. Cell Commun. Sign. CCS.

[151] Leischner H., Grundler R., Albers C., Illert A.L., Gotze K., Peschel C., Duyster J. (2010). Combination of c-SRC and FLT3 inhibitors has an additive inhibitory effect on FLT3 ITD but not on FLT3 TKD positive cells. Blood.

[152] Gozgit J.M., Wong M.J., Wardwell S., Tyner J.W., Loriaux M.M., Mohemmad Q.K., Narasimhan N.I., Shakespeare W.C., Wang F., Druker B.J. (2011). Potent activity of Ponatinib (AP24534) in models of FLT3-driven acute myeloid leukemia and other hematologic malignancies. Mol. Cancer Ther..

[153] Bourrié B., Brassard D.L., Cosnier-Pucheu S., Zilberstein A., Yu K., Levit M., Morrison J.G., Perreaut P., Jegham S., Hilairet S. (2013). SAR103168: A tyrosine kinase inhibitor with therapeutic potential in myeloid leukemias. Leuk. Lymphoma.

[154] Weir M.C., Hellwig S., Tan L., Liu Y., Gray N.S., Smithgall T.E. (2017). Dual inhibition of Fes and Flt3 tyrosine kinases potently inhibits Flt3-ITD+ AML cell growth. PLoS ONE.

[155] Weir M.C., Shu S.T., Patel R.K., Hellwig S., Chen L., Tan L., Gray N.S., Smithgall T.E. (2018). Selective inhibition of the myeloid Src-family kinase Fgr potently suppresses AML cell growth in vitro and in vivo. ACS Chem. Biol..

[156] Kentsis A., Reed C., Rice K.L., Sanda T., Rodig S.J., Tholouli E., Christie A., Valk P.J.M., Delwel R., Ngo V. (2012). Autocrine activation of the MET receptor tyrosine kinase in acute myeloid leukemia. Nat. Med..

[157] Fialin C., Larrue C., Vergez F., Sarry J.E., Bertoli S., Mansat-De Mas V., Demur C., Delabesse E., Payrastre B., Manenti S. (2013). The short form of RON is expressed in acute myeloid leukemia and sensitizes leukemic cells to cMET inhibitors. Leukemia.

[158] McGee S.F., Kornblau S.M., Qiu Y., Look A.T., Zhang N., Yoo S.Y., Coombes K.R., Kentsis A. (2015). Biological properties of ligand-dependent activation of the MET receptor kinase in acute myeloid leukemia. Leukemia.

[159] Mulgrew N.M., Kettyle L.M.J., Ramsey J.M., Cull S., Smyth L.J., Mervyn D.M., Bijl J.J., Thompson A. (2014). c-Met inhibition in a HOXA9/Meis1 model of CN-AML. Dev. Dyn..

[160] Smith C.I.E., Islam T.C., Mattsson P.T., Mohamed A.J., Nore B.F., Vihinen M. (2001). The Tec family of cytoplasmic tyrosine kinases: Mammalian Btk, Bmx, Itk, Tec, Txk and homologs in other species. Bioessays.

[161] Tang B., Mano H., Yi T., Ihle J.N. (1994). Tec kinase associates with c-kit and is tyrosine phosphorylated and activated following stem cell factor binding. Mol. Cell. Biol..

[162] Van Dijk T.B., van den Akker E., Parren-van Amelsvoort M., Mano H., Löwenberg B., von Lindern M. (2000). Stem cell factor induces phosphatidylinositol 3-kinase-dependent Lyn/Tec/Dok-1 complex formation in hematopoietic cells. Blood.

[163] Rushworth S.A., Pillinger G., Abdul-Aziz A., Piddock R., Shafat M.S., Murray M.Y., Zaitseva L., Lawes M.J., MacEwan D.J., Bowles K.M. (2015). Activity of Bruton’s tyrosine-kinase inhibitor ibrutinib in patients with CD117-positive acute myeloid leukaemia: A mechanistic study using patient-derived blast cells. Lancet Haematol..

[164] Pillinger G., Abdul-Aziz A., Zaitseva L., Lawes M., MacEwan D.J., Bowles K.M., Rushworth S.A. (2015). Targeting BTK for the treatment of FLT3-ITD mutated acute myeloid leukemia. Sci. Rep..

[165] Zaitseva L., Murray M.Y., Shafat M.S., Lawes M.J., MacEwan D.J., Bowles K.M., Rushworth S.A. (2014). Ibrutinib inhibits SDF1/CXCR4 mediated migration in AML. Oncotarget.

[166] Rushworth S.A., Murray M.Y., Zaitseva L., Bowles K.M., MacEwan D.J. (2014). Identification of Bruton’s tyrosine kinase as a therapeutic target in acute myeloid leukemia. Blood.

[167] Wu H., Hu C., Wang A., Weisberg E.L., Wang W., Chen C., Zhao Z., Yu K., Liu J., Wu J. (2016). Ibrutinib selectively targets FLT3-ITD in mutant FLT3-positive AML. Leukemia.

